# The Predictive Value of the Peripheral Arterial Calcium Scoring System on Computed Tomography Angiography in Patients With Chronic Limb-Threatening Ischemia Undergoing Below-the-Knee Endovascular Interventions

**DOI:** 10.1177/15266028251363475

**Published:** 2025-08-25

**Authors:** Michael J. Nugteren, Çağdaş Ünlü, Olaf J. Bakker, Koen M. van de Luijtgaarden, Morsal Samim, Hester J. Scheffer, Gert J. de Borst, Constantijn E.V.B. Hazenberg

**Affiliations:** 1 Department of Vascular Surgery, University Medical Center Utrecht, The Netherlands; 2 Department of Vascular Surgery, Noordwest Ziekenhuisgroep, Alkmaar, The Netherlands; 3 Department of Vascular Surgery, St. Antonius Ziekenhuis, Nieuwegein, The Netherlands; 4 Department of Vascular Surgery, Maasstad Ziekenhuis, Rotterdam, The Netherlands; 5 Department of Radiology, University Medical Center Utrecht, The Netherlands; 6 Department of Radiology, Noordwest Ziekenhuisgroep, Alkmaar, The Netherlands

**Keywords:** peripheral artery disease, CLTI, infrapopliteal, popliteal, below-the-knee, vascular calcification, PACSS

## Abstract

**Introduction:**

Endovascular below-the-knee (BTK) revascularization is safe and effective in patients with chronic limb-threatening ischemia (CLTI), but outcomes depend on the severity of limb ischemia, anatomical pattern, comorbidity, and degree of vessel calcification. The most commonly used peripheral calcification score, the peripheral arterial calcium scoring system (PACSS), is angiography based and is therefore limited by low sensitivity and interobserver agreement. This study aimed to determine the prognostic value of the original and a modified PACSS (mPACSS) based on computed tomography angiography (CTA) imaging on short-term outcomes after BTK endovascular interventions.

**Materials and Methods:**

All consecutive patients included in the prospective Dutch Chronic Lower Limb-Threatening Ischemia Registry (THRILLER) between February 2021 and July 2023 who underwent CTA imaging within 6 months before the procedure were included. The mPACSS also takes calcification of the entire target vessel into account. Primary outcomes were limb salvage and amputation-free survival (AFS) at 3 months. Secondary outcomes were technical success, primary patency, and overall survival.

**Results:**

In total, 419 patients with 473 limbs were included (mean age 74.1 ± 10.2 years; 71.1% male). PACSS 4 was present in 39.7% of the limbs and associated with higher age and higher rates of hypertension, diabetes mellitus, cardiovascular morbidity, wound, ischemia, foot infection stage, global limb anatomic staging system stage and use of stents. In multivariable analysis, PACSS 4 was significantly associated with lower limb salvage (hazard ratio [HR] 2.75, 95% confidence intervals [CI] 1.49-5.08, *P* = .001) and AFS (HR 1.64, 95% CI 1.07-2.53, *P* = .025), as was mPACSS ≥5 (limb salvage: HR 2.22, 95% CI 1.88-5.46, *P* = .015; AFS: HR 1.68, 95% CI 1.06-2.65, *P* = .026). No significant differences were found in terms of technical success, primary patency, and overall survival.

**Conclusion:**

Both PACSS and mPACSS scored on CTA imaging are significantly associated with 3-month limb salvage and AFS after BTK endovascular interventions.

**Clinical Impact:**

Current peripheral calcification scores are based on angiography and limited by a low sensitivity and minimal interobserver agreement. Scoring the original and modified peripheral arterial calcium scoring system (mPACSS) on computed tomography angiography (CTA) has proven to be more reliable and fast. Accordingly, this study shows that both PACSS and mPACSS scored on CTA are independent predictors of 3-month outcomes after below-the-knee interventions. This reinforces the importance of using a reliable peripheral calcification score in the preoperative assessment of CLTI patients eligible for revascularization. Future evaluations in larger cohorts with longer follow-up should investigate the PACSS and mPACSS in different clinical contexts and whether either score is superior.

## Introduction

Peripheral calcification forms one of the main challenges in endovascular revascularization for peripheral arterial disease (PAD).^1^ In the coming decades, the increase in the global prevalence of diabetes mellitus (DM) is expected to cause a similar increase in the prevalence of PAD and complexity of lesions due to peripheral calcification.^2,3^

Chronic limb-threatening ischemia (CLTI), the end stage of PAD, is associated with high amputation and mortality rates if left untreated.^4^ Endovascular below-the-knee (BTK) revascularization is safe and effective in patients with CLTI.^5,6^ However, outcomes strongly depend on the severity of limb ischemia, anatomical pattern, and degree of vessel calcification.^1,5^

In the field of coronary artery disease (CAD), the quantitative coronary artery calcium score has been proven to be an accurate predictor of short- and long-term outcomes, especially after percutaneous coronary intervention.^7-9^ As a result, this score is currently used worldwide for risk stratification in clinical practice.^7^

In the field of PAD, multiple quantitative and semiquantitative calcium scores have been developed.^10-20^ Higher calcification grades were negatively associated with technical success, patency, target lesion revascularization (TLR), major adverse cardiovascular events (MACE), major amputation, and mortality after endovascular revascularization.^10-20^ However, the most commonly used peripheral calcification scores, the semiquantitative peripheral arterial calcium scoring system (PACSS), and peripheral academic research consortium are scored on 2-dimensional angiographic imaging. Previous studies found low sensitivity and interobserver agreement for calcification scores based on angiography.^21,22^ In contrast, scoring PACSS on computed tomography angiography (CTA) is proven to be reliable in popliteal and infrapopliteal disease.^23^ Lastly, previous studies have shown that calcification of the entire infrapopliteal target vessel (TV) may be relevant in predicting outcomes rather than target lesion (TL) calcification alone.^11,20^

The aim of this study was to determine the prognostic value of the original and a modified CTA-based PACSS score on short-term clinical outcomes after popliteal and infrapopliteal endovascular interventions for CLTI.

## Materials and Methods

The DuTcH chRonIc Lower Limb-threatening ischEmia Registry (THRILLER) is a national multicenter prospective registry that includes all consecutive patients that undergo a popliteal or infrapopliteal endovascular intervention in 7 Dutch hospitals. The protocol was approved by the local ethics committees and informed consent was obtained from all patients. The study protocol and short-term outcomes were previously published.^5,24^

For this analysis, all patients who were treated between February 2021 and July 2023 and underwent a CTA scan within 6 months before the index procedure were extracted. According to the global vascular guidelines (GVG) for CLTI, vascular imaging should be performed in all patients with suspected CLTI to determine the presence, extent, and severity of arterial disease and to help informed decisions about revascularization. For those with tibial disease, particularly in the setting of tissue loss, CTA and magnetic resonance angiography (MRA) may offer useful information. CTA offers a sensitivity of 95% and specificity of 91% in the infrapopliteal segment.^25^ Whether a CTA scan had been performed in THRILLER depended on center and physician preference, indication, and the presence of recent imaging.

Exclusion criteria were patients with acute limb ischemia, BTK interventions as a result of distal embolization, CTA scans with insufficient quality to score calcification length or circumference, and patients unable to give informed consent. Data regarding patient demographics, comorbidities, medical examinations and imaging, lesion characteristics, procedural characteristics, follow-up, and outcomes were collected from electronic medical records and entered into an online data capture software. The wound, ischemia, foot infection (WIfI), and global limb anatomic staging system (GLASS) scores were graded according to the GVG for CLTI.^25^

## Interventions and Follow-Up

Patients were included after an endovascular intervention of the popliteal and tibial arteries, regardless of technical success, simultaneous proximal (hybrid) interventions, or simultaneous amputations. Interventions were performed by a vascular surgeon or interventional radiologist. The selection of guidewires, balloons, stents, other endovascular devices, and postoperative antithrombotic strategy were at the discretion of the interventionalist. Optimal medical therapy was recommended in all patients. The standardized follow-up consisted of clinical examination, resting systolic toe pressure, and duplex ultrasonography (DUS) at 6 to 8 weeks. The rest of the follow-up was at the discretion of the treating physician.

## (Modified) Peripheral Arterial Calcium Scoring System

**Figure 1.**
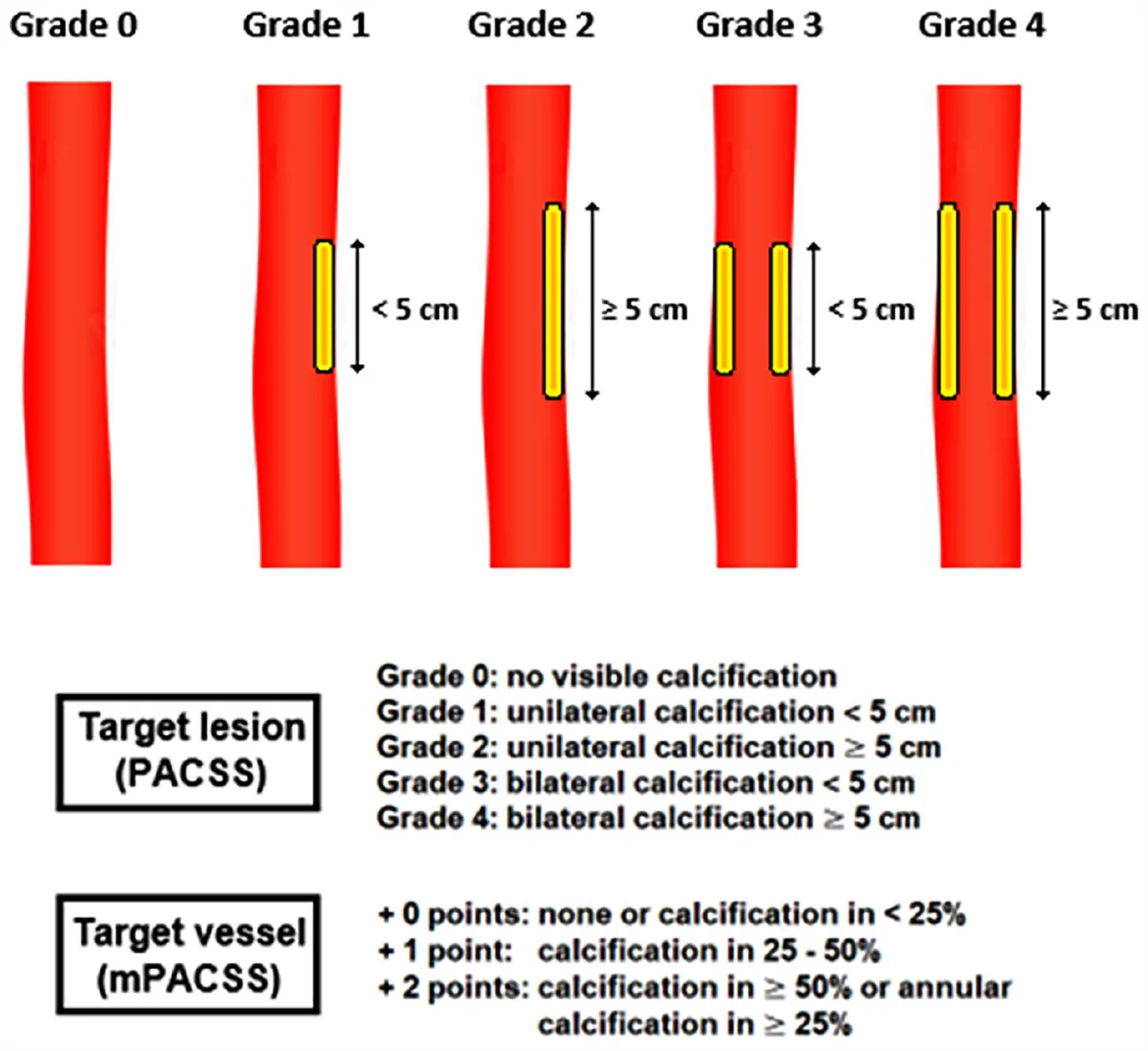
The peripheral arterial calcium scoring system and its modification. The original PACSS score consists of 5 grades based on the TL calcification. The mPACSS score also takes into account calcification in the remaining TV. The TV is the POP, ATA, PTA or PA. In the case, the TL is located in both the popliteal and an infrapopliteal artery, both arteries are considered the TV. In the case, the TL is located solely in the TPT, the TV includes the TPT and the PTA or PA, depending on the target arterial path. ATA, anterior tibial; mPACSS, modified PACSS; PA, peroneal artery; PACSS, peripheral arterial calcium scoring system; POP, popliteal; PTA, posterior tibial; TL, target lesion; TPT, tibioperoneal trunk; TV, target vessel.

All lesions were scored according to the PACSS score based on the preoperative CTA scan. The PACSS score grades circumference (unilateral vs bilateral) and length (5 cm cut-off) of vessel calcification, grading lesions from PACSS 0 to PACSS 4 (Figure 1).

Because studies have demonstrated that calcification of the entire TV is relevant to predict outcomes, a modification was made to the original PACSS score.^11,20^ In this modification, total TV calcification is scored as a percentage of total TV length, resulting in a TV score of 0 to 2 (Figure 1, lower panel). The modified PACSS (mPACSS) score consists of the sum of the original PACSS score and the TV score, ultimately giving patients a mPACSS score of 0 to 6. The inter- and intraobserver agreement of both PACSS and mPACSS have been demonstrated to be moderate to almost perfect.^23^ Possible TVs are the popliteal (POP), anterior tibial (ATA), posterior tibial (PTA), or peroneal artery (PA). In the case, the TL is located solely in the tibioperoneal trunk (TPT), the TV includes the TPT and the PTA or PA, depending on the target arterial path. In the case that multiple lesions were treated in 1 limb, the highest PACSS and mPACSS score was used for further analysis.

## CTA and Scoring Procedures

On perioperative angiography, the culprit lesions which had been treated were localized and correlated with and segmented using the preoperative CTA scan. CT images were acquired using a 64- or 128-slice Ingenuity CT scanner (Philips Healthcare, Best, The Netherlands) and reconstructed using iterative model reconstruction 6/1 (IMR; Philips Healthcare, Best, The Netherlands). CTA scans were performed using 98 to 146 mAs, 120 kV, window width/window level (WW/WL) 600/150 with a postthreshold delay of 12 seconds. Contrast volume varied from 60 to 100 ml. High-resolution thin-slice reconstructions (0.9 or 1.5 mm slice thickness) were used to allow detailed assessment of calcification in (infra)popliteal arteries. Use of bone window setting further enhanced calcium visualization.

PACSS and mPACSS were scored by 1 rater (M.N.) according to predefined arrangements (Table 1). Solitary lesions in the most distal one-third of the infrapopliteal arteries were not scored because assessing the circumference in these segments was assumed not to be reliable. In the case of doubt, a second rater (C.H.) was consulted to reach consensus.

**Table 1.** Arrangements in Assessing the (Modified) PACSS Score on CTA Imaging.

Section	Arrangements
General	1. Only popliteal and tibial arteries will be scored (until the ankle joint). Solitary lesions in the most distal one-third of the tibial arteries will not be scored.
	2. If available, use transverse images to determine the length and circumference of the calcification.
TL	3. The length of the TL equals the length of contiguous stenosis in the peripheral arteries (defined as percent stenosis of diameter >50%).
	4. Calcification in the TV on a different level than the TL does not count as lesion calcification.
	5. In line with arrangement 4, the length of TL calcification cannot exceed the TL length, that is, lesions <5 cm cannot be scored as grade 2 or 4.
	6. If the TL consists of multiple calcified plaques, add the lengths of the individual calcified plaques together to determine if the TL calcification is longer than 5 cm.
	7. Spotty calcification counts in general as grade 1.
Circumference	8. If only a small part of the treated lesion is bilateral, the entire lesion will be scored as bilateral.
	9. A bilateral lesion does not necessarily have to be annular.
TV	10. In the case, the TL is located in both the popliteal and an infrapopliteal arteries, and both arteries are considered the TV.
	11. In the case, the TL is located solely in the TPT, the TV includes the TPT and the PTA or PA, depending on the target arterial path.
	12. If the TV consists of multiple calcified plaques, add the lengths of the individual calcified plaques together to determine the length of TV calcification.

Abbreviations: CTA, computed tomography angiography; PA, peroneal artery; PACSS, peripheral arterial calcium scoring system; PTA, posterior tibial; TL, target lesion; TPT, tibioperoneal trunk; TV, target vessel.

### Study Outcomes and Definitions

The primary outcomes were limb salvage and amputation-free survival (AFS) at 3 months. Limb salvage was defined as freedom from major amputation (above the ankle), while AFS was defined as freedom from death and major amputation.

Secondary outcomes were technical success, primary patency at the 6 to 8 week visit and overall survival at 3 months. Technical success was defined as a residual diameter stenosis <50% for angioplasty and <30% for stenting and in-line flow to the foot at the end of the procedure if intended. Primary patency was defined as freedom from a restenosis >50%, as measured on MRA, CTA, digital subtraction angiography, or DUS with a peak systolic velocity-ratio of >2.4. Overall survival was defined as freedom from death from any cause.

In the case of ambiguity or controversy, an endpoint was assessed by a clinical adjudication committee, previously declared in THRILLER, consisting of at least 3 vascular specialists.

Outcomes were distributed per PACSS score, mPACSS score, and TV score. In addition, these scores were dichotomized based on different cut-off values, such as length or annularity, to achieve the highest predictive value.

## Statistical Analysis

All statistical analyses were performed with IBM SPSS Statistics (version 29.0. Armonk, NY, USA) and RStudio (version 1.3. Boston, MA, USA). Continuous variables are presented as mean value ± standard deviation. Categorical variables, technical success, and primary patency are presented as absolute number and proportion of the study population. Baseline characteristics were compared using the chi-squared test for categorical variables and the independent samples *t*-test or Mann–Whitney *U* test for continuous variables, depending on the distribution, which was assessed using histograms. Technical success and primary patency were compared with binary logistic regression. Primary patency was only calculated for patients with technical success and available imaging at 6 to 8 weeks. Limb salvage, AFS, and overall survival were estimated with the Kaplan–Meier method and differences between groups were compared with the log-rank test. Statistical significance was set at *P* < .05.

Univariable and multivariable Cox proportional hazards models were used to explore whether PACSS and mPACSS score were independent predictors of outcomes and to calculate hazard ratios (HR) and 95% confidence intervals (CI). Patients with missing data were removed from this analysis. The multivariable adjusted model was made with a backward selection method starting with variables with a *P*-value < .1 in the univariable model and removing variables one by one based on the highest *P*-value. Only variables with a *P*-value < .05 were kept in the final model. The proportional hazards assumption was assessed through visual inspection of log-minus-log survival plots and analysis of Schoenfeld residuals.

## Results

### Baseline Characteristics

In total, 840 patients with 947 limbs were treated during the study period. Of these, 419 patients with 473 limbs were eligible for inclusion due to the presence of a CTA scan within 6 months before the index procedure. PACSS 4 was present in 39.7% of the included limbs.

The baseline characteristics are summarized in Table 2. Patients with PACSS 4 were older (75.6 ± 9.3 vs 73.2 ± 10.6, *P* = .018) and featured higher rates of male sex (77.2% vs 67.3%, *P* = .030), hypertension (95.1% vs 88.7%, *P* = .026), DM (71.0% vs 56.0%, *P* = .002), CAD (56.8% vs 43.2%, *P* = .007) and congestive heart failure (25.9% vs 16.7%, *P* = .023). In addition, limbs with PACSS 4 presented with higher Rutherford categories (*P* = .044), higher WIfI Wound grade (*P* = .007), higher WIfI foot infection grade (*P* = .011), and higher WIfI stage (*P* = .003), but similar WIfI Ischemia grade (*P* = .547).

**Table 2.** Baseline Characteristics.

Patients	All (n = 419)	PACSS 0-3 (n = 257)	PACSS 4 (n = 162)	*P*-value
Limbs	All (n = 473)	PACSS 0-3 (n = 285)	PACSS 4 (n = 188)	*P*-value
Mean age, years	74.1 ± 10.2	73.2 ± 10.6	75.6 ± 9.3	**.018**
Male	298 (71.1)	173 (67.3)	125 (77.2)	**.030**
Hypertension	382 (91.2)	228 (88.7)	154 (95.1)	**.026**
Hyperlipidemia	359 (85.7)	215 (83.7)	144 (88.9)	.137
Diabetes mellitus	259 (61.8)	144 (56.0)	115 (71.0)	**.002**
History of smoking (n = 413)				
Current	100 (23.9)	69 (27.1)	31 (19.6)	.094
Previous	229 (54.7)	131 (51.4)	98 (62.0)	
Coronary artery disease	203 (48.4)	111 (43.2)	92 (56.8)	**.007**
Congestive heart failure	85 (20.3)	43 (16.7)	42 (25.9)	**.023**
Cerebrovascular disease	106 (25.3)	61 (23.7)	45 (27.8)	.354
Renal insufficiency (GFR < 30 ml/min)	39 (9.3)	27 (10.5)	12 (7.4)	.288
Previous TL revascularization	156 (33.0)	93 (32.6)	63 (33.5)	.842
Previous TL amputation	51 (10.8)	31 (10.9)	20 (10.6)	.935
Rutherford category				
4	57 (12.1)	43 (15.1)	14 (7.4)	**.044**
5	396 (83.7)	233 (81.8)	167 (88.8)	
6	20 (4.2)	9 (3.2)	7 (3.7)	
WIfI wound grade				
0	57 (12.1)	43 (15.1)	14 (7.4)	**.007**
1	167 (35.3)	109 (38.2)	58 (30.9)	
2	229 (48.4)	123 (43.2)	106 (56.4)	
3	20 (4.2)	10 (3.5)	10 (5.3)	
WIfI ischemia grade (n = 429)^a^				
0	56 (13.1)	38 (14.6)	18 (10.7)	.547
1	102 (23.8)	62 (23.8)	40 (23.7)	
2	100 (23.3)	56 (21.5)	44 (26.0)	
3	171 (39.9)	104 (40.0)	67 (39.6)	
WIfI foot infection grade				
0	257 (54.3)	171 (66.5)	86 (45.7)	**.011**
1	52 (11.0)	31 (10.9)	21 (11.2)	
2	139 (29.4)	72 (25.3)	67 (35.6)	
3	25 (5.3)	11 (3.9)	14 (7.4)	
WIfI stage (n = 429)^a^				
1	69 (16.1)	50 (19.2)	19 (11.2)	**.003**
2	75 (17.5)	53 (20.4)	22 (13.0)	
3	97 (22.6)	60 (23.1)	37 (19.7)	
4	188 (43.8)	97 (37.3)	91 (48.4)	

Continuous data are presented as the mean value ± standard deviation. Categorical data are presented as the counts (percentage). Bold indicates statistical significance (*p* < 0.05).

Abbreviations: GFR = glomerular filtration rate; PACSS = peripheral arterial calcium scoring system; TL = target limb; WIfI = wound, ischemia, foot infection.

a In 44 patients, the WIfI ischemia stage could not be calculated due to previous minor amputations or the absence of both a toe pressure and ankle-brachial index.

The lesion characteristics are summarized in Table 3. Lesions with PACSS 4 featured a longer mean lesion length (176.2 ± 98.4 vs 85.6 ± 100.1, *P* < .001), more total occlusions (69.7% vs 40.9%, *P* < .001), and consequently a higher GLASS stage (*P* < .001). Additionally, bare metal stents (BMS; 17.9% vs 9.9%, *P* = .002) and drug-eluting stents (DES; 5.1% vs 1.3%, *P* = .001) were more often used in PACSS 4 lesions.

**Table 3.** Lesion Characteristics.

Lesions	All (n = 779)	PACSS 0-3 (n = 545)	PACSS 4 (n = 234)	*P*-value
Technical success	707 (90.8)	511 (93.8)	196 (83.8)	**<.001**
Lesion location				
Popliteal	213 (27.3)	147 (27.0)	66 (28.2)	.933
Popliteal-tibial	46 (5.9)	32 (5.9)	14 (6.0)	
Tibial	520 (66.8)	366 (67.2)	154 (65.8)	
Target lesion length, mm	112.8 ± 107.8	85.6 ± 100.1	176.2 ± 98.4	**<.001**
Total occlusions	386 (49.6)	223 (40.9)	163 (69.7)	**<.001**
GLASS stage				
1	125 (16.0)	125 (22.9)	0 (0)	**<.001**
2	143 (18.4)	122 (22.4)	21 (9.0)	
3	158 (20.3)	118 (21.7)	40 (17.1)	
4	353 (45.3)	180 (33.0)	173 (73.9)	
Endovascular devices (n = 707)				
POBA alone	605 (85.6)	408 (74.9)	128 (54.7)	**<.001**
DCB	51 (7.2)	38 (7.0)	13 (5.6)	.464
BMS	96 (13.6)	54 (9.9)	42 (17.9)	**.002**
DES	19 (2.7)	7 (1.3)	12 (5.1)	**.001**
Atherectomy	3 (0.4)	2 (0.4)	1 (0.4)	.901
IVL	32 (4.5)	20 (3.7)	12 (5.1)	.422

Continuous data are presented as the mean value ± standard deviation. Categorical data are presented as the counts (percentage). Bold indicates statistical significance (*p* < 0.05).

Abbreviations: BMS, bare metal stenting; DCB, drug-coated balloon; DES, drug-eluting stent; GLASS, global limb anatomic staging system; IVL, intravascular lithotripsy; POBA, plain old balloon angioplasty; SFA, superficial femoral artery.

### Primary Outcomes

**Figure 2.**
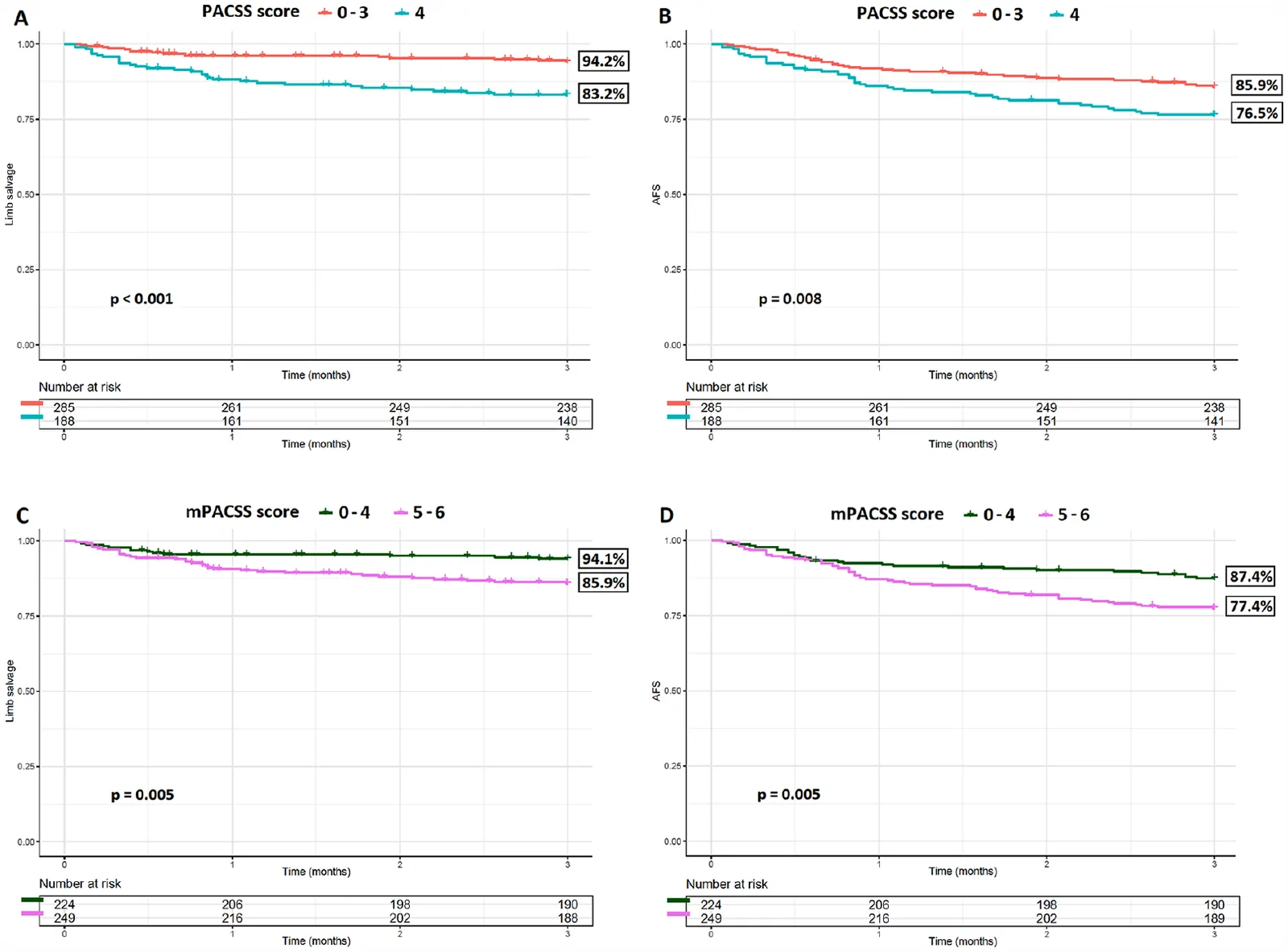
Kaplan–Meier curves of the limb salvage and AFS distributed per PACSS score (A, B) and mPACSS score (C, D). AFS, amputation-free survival; PACSS, peripheral arterial calcium scoring system; mPACSS, modified PACSS.

The primary outcomes of different cut-off values of the PACSS, mPACSS, and TV score are shown and summarized in Figure 2 and Supplemental Table 1. Regarding the original PACSS score, PACSS 4 versus PACSS 0 to 3 showed the lowest predictive value for limb salvage (83.2% vs 94.2%, *P* < .001) and AFS (76.5% vs 85.9%, *P* = .008). Regarding the mPACSS score, mPACSS 5 to 6 versus 0 to 4 showed the lowest predictive value for limb salvage (85.9% vs 94.1%, *P* = .005) and AFS (77.4% vs 87.4%, *P* = .005).

The univariable and multivariable models for limb salvage and AFS are shown in Tables 4 and 5. In the multivariable model for major amputation (= 1 − limb salvage), technical failure (HR 5.60, 95% CI 2.57-12.22, *P* < .001), renal insufficiency (HR 3.36, 95% CI 1.64-6.88, *P* < .001), Rutherford category 6 (HR 25.98, 95% CI 3.27-106.29, *P* = .002), and PACSS 4 (HR 2.75, 95% CI 1.49-5.08, *P* = .001) or mPACSS ≥5 (HR 2.22, 95% CI 1.17-4.22, *P* = .015) were independent predictors. In the multivariable model for major amputation or death (=1 − AFS), technical failure (HR 4.44, 95% CI 2.33-8.46, *P* < .001), renal insufficiency (HR 3.21, 95% CI 1.88-5.46, *P* < .001), Rutherford categories 5 (HR 4.16, 95% CI 1.02-17.02, *P* = .048), and 6 (HR 21.44, 95% CI 4.75-96.79, *P* < .001), and PACSS 4 (HR 1.64, 95% CI 1.07-2.53, *P* = .025) or mPACSS ≥5 (HR 1.68, 95% CI 1.06-2.65, *P* = .026) were independent predictors.

**Table 4.** Hazard Ratios for Major Amputation at 3 Months.

	Univariate		Multivariable model, PACSS 0-3 vs 4		Multivariable model, mPACSS 0-4 vs 5-6	
Variables	HR (95% CI)	*P*-value	HR (95% CI)	*P*-value	HR (95% CI)	*P*-value
Technical failure	5.16 (2.41-11.04)	<**.001**	5.60 (2.57-12.22)	<.001	6.00 (2.76-13.09)	<.001
Age >75 = 1	0.79 (0.45-1.41)	.424				
Male = 1	1.26 (0.64-2.48)	.498				
Hypertension	4.83 (0.67-35.05)	.119				
Hyperlipidemia	1.34 (0.53-3.38)	.538				
Diabetes mellitus	1.99 (1.01-3.91)	**.046**				
Current smoker	1.16 (0.60-2.24)	.654				
Coronary artery disease	1.01 (0.57-1.78)	.984				
Congestive heart failure	1.35 (0.71-2.56)	.358				
Renal insufficiency (GFR < 30 ml/min)	2.75 (1.37-5.53)	**.005**	3.36 (1.64-6.88)	<.001	2.80 (1.37-5.70)	.005
Cerebrovascular disease	1.55 (0.85-2.84)	.153				
Previous target limb revascularization	1.48 (0.83-2.63)	.186				
Previous or concomitant target limb amputation	2.21 (1.25-3.92)	**.007**				
Rutherford category (vs 4)		**<.001**		<.001		<.001
5	5.41 (0.74-39.47)	.096	3.78 (0.52-27.69)	.191	3.85 (0.52-28.29)	.185
6	40.6 (5.19-317.73)	<.001	25.98 (3.27-206.29)	.002	32.92 (4.19-258.84)	<.001
GLASS score (vs 1)		.299				
2	2.56 (0.32-20.79)	.380				
3	2.70 (0.34-21.31)	346				
4	4.13 (0.56-30.30)	.163				
PACSS score ≥3	2.21 (1.07-4.57)	**.033**				
PACSS score 2 or 4 (>5 cm)	2.94 (1.61-5.37)	<**.001**	2.75 (1.49-5.08)	.001	2.22 (1.17-4.22)	.015
PACSS 4	3.08 (1.69-5.64)	<**.001**				
mPACSS ≥4	2.44 (1.22-4.92)	**.012**				
mPACSS ≥5	2.44 (1.29-4.63)	**.006**				

Different cut-off values of PACSS are tested in the univariate model to explore the highest level of significance. Bold indicates statistical significance (*p* < 0.05).

Abbreviations: CI, confidence interval; GFR, glomerular filtration rate; GLASS, global limb anatomic staging system; HR, hazard ratio; (m)PACSS, (modified) peripheral arterial calcium scoring system.

**Table 5.** Hazard Ratios for Major Amputation or Death (1 − AFS) at 3 Months.

	Univariate		Multivariable model: PACSS 0-3 vs 4		Multivariable model: mPACSS 0-4 vs 5-6	
Variables	Hazard ratio (95% CI)	*P*-value	HR (95% CI)	*P*-value	Hazard ratio (95% CI)	*P*-value
Technical failure	3.91 (2.07-7.37)	< **.001**	4.44 (2.33-8.46)	<.001	4.45 (2.34-8.49)	<.001
Age >75 = 1	1.38 (0.89-2.13)	.148				
Male = 1	1.15 (0.70-1.89)	.572				
Hypertension	2.88 (0.91-9.13)	**.072**				
Hyperlipidemia	1.33 (0.66-2.66)	.419				
Diabetes mellitus	1.52 (0.95-2.44)	**.082**				
Current smoker	0.92 (0.55-1.56)	.765				
Coronary artery disease	1.21 (0.79-1.86)	.384				
Congestive heart failure	1.42 (0.88-2.28)	.148				
Renal insufficiency (GFR < 30 ml/min)	2.85 (1.69-4.80)	<**.001**	3.21 (1.88-5.46)	<.001	2.93 (1.72-4.97)	<.001
Cerebrovascular disease	1.07 (0.66-1.73)	.797				
Previous target limb revascularization	0.99 (0.63-1.56)	.976				
Previous or concomitant target limb amputation	1.76 (1.15-2.71)	**.010**				
Rutherford category (vs 4)		**<.001**		<.001		<.001
5	5.30 (1.30-21.60)	.020	4.16 (1.02-17.02)	.048	4.12 (1.01-16.88)	.049
6	26.38 (5.90-118.04)	<.001	1.44 (4.75-96.79)	<.001	23.26 (5.18-104.46)	<.001
GLASS score (vs 1)		.328				
2	1.85 (0.54-6.39)	.331				
3	1.62 (0.47-5.54)	.446				
4	2.34 (0.73-7.49)	.154				
PACSS score ≥3	1.68 (1.02-2.78)	**.043**				
PACSS score 2 or 4 (>5 cm)	1.68 (1.10-2.58)	**.018**	1.64 (1.07-2.53)	.025	1.68 (1.06-2.65)	.026
PACSS 4	1.77 (1.15-2.71)	**.009**				
mPACSS ≥4	1.76 (1.09-2.85)	**.021**				
mPACSS ≥5	1.88 (1.20-2.96)	**.006**				

Different cut-off values of PACSS are tested in the univariate model to explore the highest level of significance. Bold indicates statistical significance (*p* < 0.05).

Abbreviations: AFS, amputation-free survival; CI, confidence interval; GFR, glomerular filtration rate; GLASS, global limb anatomic staging system; (m)PACSS, (modified) peripheral arterial calcium scoring system.

### Secondary Outcomes

The secondary outcomes are summarized in Figure 2 and Supplemental Tables 2 to 4. In univariable analysis, PACSS 4 lesions were significantly associated with lower technical success (83.8% vs 93.8%, *P* < .001), but not with lower primary patency (74.4% vs 81.9%, *P* = .071). No dichotomized mPACSS score resulted in significant differences in terms of technical success and primary patency, nor was PACSS or mPACSS significantly associated with overall survival.

The univariable and multivariable models for technical success and primary patency are shown in Figure 2 and Supplemental Tables 3 and 4. In the multivariable models, PACSS and mPACSS were not independent predictors of technical success or primary patency.

## Discussion

Both PACSS 4 and mPACSS ≥5 are independent predictors of decreased limb salvage and AFS 3 months after endovascular revascularization. No superiority of either PACSS or mPACSS was demonstrated in this study.

Previous studies correlating PACSS and follow-up outcomes scored calcification in the femoropopliteal artery on angiography and found higher PACSS grades to be independent predictors of loss of patency, major adverse limb events, mortality, and TLR.^13-17^ Only one previous study scored PACSS in infrapopliteal arteries and found PACSS 4 to be associated in univariable analysis with unsuccessful guidewire crossing of BTK chronic total occlusions.^26^ The majority of aforementioned studies primarily included patients with intermittent claudication and therefore did not analyze or find a correlation between PACSS and limb salvage or AFS. Our study scored PACSS in the popliteal and infrapopliteal arteries of CLTI patients and made multiple comparisons to determine the optimal cut-off value.

Dichotomizing PACSS scores is justifiable because in clinical practice, the interventionalist is generally confronted with binary questions regarding treatment options, namely if the calcification is severe enough to convert to additional calcium modifying treatment modalities such as atherectomy or specialty balloons.^27,28^ Four of the 7 previous PACSS studies found PACSS 4 to be predictive, 2 studies used a cut-off value of PACSS ≥3, and 1 study used a cut-off value of PACSS ≥2. Although differences were sometimes small between different cut-off values in this study, PACSS 0 to 3 versus 4 and mPACSS 0 to 4 versus 5 to 6 achieved the highest significant levels in terms of limb salvage, AFS, and technical success (Tables 4 and 5; Figure 2 and Supplemental Table 1).

PACSS, as well as other commonly used peripheral calcification scores, was originally designed to be scored on angiographic imaging. However, angiography seems not to be the optimal modality to quantify calcification. Two previous studies found that the sensitivity of angiography for detecting calcium in the femoropopliteal arteries was only 59% to 76% as compared to intravascular ultrasound.^21,22^ Moreover, the inter- and intraobserver agreement of a binary PACSS score on catheter based angiography (PACSS 0-2 vs 3-4) was fair (κ = 0.32) and moderate to substantial (κ = 0.52-0.62) according to the scale recommended by Landis and Koch, respectively.^21^ On the contrary, 1 study found that the inter- and intraobserver agreement of PACSS scoring on CTA imaging was moderate (κ = 0.60) and almost perfect (κ = 0.86).^23^ In addition, CTA scoring has the advantages of being 3-dimensional and noninvasive, and can be performed preoperatively for risk stratification. In conclusion, the use of CTA imaging to score the semi-quantitative PACSS and mPACSS scores is imperative for reliable and accurate scoring.

Regarding the correlation between the quantity of calcification and clinical outcome, 2 studies found that calcification of the entire infrapopliteal TV was predictive for technical success, limb salvage, AFS, and MACE.^11,20^ Since calcification of the entire TV might be a more accurate representation of total limb calcification than TL calcification alone, we decided to score both TL and TV calcification, leading to the mPACSS score. Nevertheless, we found no superiority of the PACSS, mPACSS, or TV calcification score in this study. Validation of these scores in a cohort with longer follow-up may shed more light on whether either score is superior.

Scoring PACSS and mPACSS on CTA imaging has proven to be reliable, fast, and independently predictive for the most important short-term outcomes after popliteal and infrapopliteal endovascular interventions. In the current GVG for CLTI, patient selection is based on the patient risk, limb severity, and anatomic complexity (PLAN) approach. Anatomic complexity is scored with GLASS, which includes a subjective dichotomous calcification score due to the lack of an easy-to-use validated calcification score. PACSS and mPACSS have potential to address this lack and refine the PLAN approach.

Limitations of this study are the nonrandomized design, the specific study population, relatively short follow-up, and adherence to the follow-up protocol. The nonrandomized design of the study resulted in clinically relevant differences in baseline and lesion characteristics. However, these differences are inevitable when comparing groups with different degrees of calcification and were addressed by the use of multivariable regression analyses. Nevertheless, the number of relevant variables exceeded what could feasibly be included in the multivariable analysis, resulting in the exclusion of certain variables such as differences in medication use, including antithrombotic therapy. The study population consisted of CLTI patients that underwent BTK endovascular interventions, which limit the generalizability of the findings to the overall PAD population. Because the 3-month follow-up of this study is relatively short, the analysis might have been underpowered to show associations in the secondary outcomes, such as primary patency and overall survival. Also, not all lesions could be scored for primary patency due to the absence of imaging at the 6 to 8 week visit in 37.7% of the lesions. On the other hand, this study used data from the well-designed and well-characterized multicenter THRILLER registry and currently represents the largest study investigating the predictive value of peripheral arterial calcification on clinical outcomes.

Regarding PACSS and mPACSS on CTA imaging, there are also some limitations to discuss. First, scoring arrangements were imperative to ensure reliability, but may have conflicted with the original assignment of PACSS grades. For example, lesions smaller than 5 cm could not be scored in grades 2 and 4, resulting in only 5 limbs being scored as PACSS grade 2. Nevertheless, previous studies described similarly low rates of PACSS grade 2 (1.1%-11.9%), despite scoring PACSS in femoropopliteal lesions on angiography imaging.^13-18^ Second, the dependency on CTA availability may hamper generalizability of the (m)PACSS score. Lastly, the limitations related to CTA imaging, such as ionizing radiation and potential contrast-induced nephrotoxicity, should be noted.

Future research should focus on testing the reliability of CTA based (m)PACSS on femoropopliteal disease and validating these scores on mid- and long-term follow-up outcomes. In addition, previous studies have shown that quantitative scoring of peripheral calcification on CTA imaging is reliable and predictive for technical success, limb salvage, AFS, and MACE.^11,20^ In clinical practice, quantitative and semi-quantitative calcification scores can be simultaneously useful because of global differences in availability of imaging modalities, software, and physician expertise. Therefore, further development of quantitative calcification scoring, including (semi-)automatic segmentation, should also be a focus of future research.

## Conclusion

PACSS and mPACSS scored on CTA imaging are significantly associated with limb salvage and AFS 3 months after BTK endovascular interventions. No superiority was found of either score.

## Supplemental Material

sj-docx-1-jet-10.1177_15266028251363475 – Supplemental material for The Predictive Value of the Peripheral Arterial Calcium Scoring System on Computed Tomography Angiography in Patients With Chronic Limb-Threatening Ischemia Undergoing Below-the-Knee Endovascular Interventions Supplemental material, sj-docx-1-jet-10.1177_15266028251363475 for The Predictive Value of the Peripheral Arterial Calcium Scoring System on Computed Tomography Angiography in Patients With Chronic Limb-Threatening Ischemia Undergoing Below-the-Knee Endovascular Interventions by Michael J. Nugteren, Çağdaş Ünlü, Olaf J. Bakker, Koen M. van de Luijtgaarden, Morsal Samim, Hester J. Scheffer, Gert J. de Borst and Constantijn E.V.B. Hazenberg in Journal of Endovascular Therapy
