# Supplementary material for: The Predictive Value of the Peripheral Arterial Calcium Scoring System on Computed Tomography Angiography in Patients With Chronic Limb-Threatening Ischemia Undergoing Below-the-Knee Endovascular Interventions
Source: J Endovasc Ther. 2025 Aug 25;33(5):2609–20. doi: 10.1177/15266028251363475 (PMC13571001; doi:10.1177/15266028251363475)
Supplement: Supplementary file 1 [file sj-docx-1-jet-10.1177_15266028251363475.docx]

**Supplementary Table 1. Kaplan Meier estimates of limb salvage, amputation-free survival (AFS) and overall survival at 3 months.**

| **PACSS** | **Patients / Limbs** | **Limb salvage** | | **AFS** | | **Overall survival** | |
| --- | --- | --- | --- | --- | --- | --- | --- |
|  |  | Estimate | p-value | Estimate | p-value | Estimate | p-value |
| **0** | 44 / 50 | 91.6% | **.004** | 83.7% | .084 | 90.6% | .924 |
| **1** | 97 / 104 | 95.0% |  | 88.4% |  | 92.7% |  |
| **2** | 5 / 5 | 100% |  | 100% |  | 100% |  |
| **3** | 111 / 126 | 94.2% |  | 84.1% |  | 90.1% |  |
| **4** | 162 / 188 | 83.2% |  | 76.5% |  | 90.7% |  |
|  | | | | | | | |
| **0-2 (unilat)** | 146 / 159 | 94.1% | **.028** | 87.3% | **.040** | 92.4% | .519 |
| **3-4 (bilat)** | 272 / 313 | 87.5% |  | 79.5% |  | 90.4% |  |
|  | | | | | | | |
| **0,1,3 (< 5cm)** | 252 / 280 | 94.1% | **< .001** | 85.6% | **.016** | 91.2% | .970 |
| **2,4 (≥ 5 cm)** | 167 / 193 | 83.6% |  | 77.1% |  | 90.9% |  |
|  | | | | | | | |
| **0-3** | 257 / 285 | 94.2% | **< .001** | 85.9% | **.008** | 91.4% | .845 |
| **4** | 162 / 188 | 83.2% |  | 76.5% |  | 90.7% |  |
|  | | | | | | | |
| **mPACSS** | **Patients / Limbs** | **Limb salvage** | | **AFS** | | **Overall survival** | |
|  |  | Estimate | p-value | Estimate | p-value | Estimate | p-value |
| **0** | 44 / 50 | 91.6% | .052 | 83.7% | .183 | 90.6% | .479 |
| **1** | 89 / 96 | 94.6% |  | 87.4% |  | 92.1% |  |
| **2** | 4 / 4 | 100% |  | 100% |  | 100% |  |
| **3** | 32 / 34 | 97.1% |  | 91.2% |  | 93.8% |  |
| **4** | 34 / 40 | 92.5% |  | 87.4% |  | 97.1% |  |
| **5** | 75 / 83 | 90.8% |  | 78.3% |  | 85.3% |  |
| **6** | 141 / 166 | 83.4% |  | 77.0% |  | 91.4% |  |
|  | | | | | | | |
| **0-2** | 137 / 150 | 93.8% | .055 | 86.5% | .097 | 91.8% | .726 |
| **3-6** | 282 / 323 | 87.9% |  | 80.1% |  | 90.7% |  |
|  | | | | | | | |
| **0-3** | 169 / 184 | 94.4% | **.009** | 87.4% | **.019** | 92.2% | .519 |
| **4-6** | 250 / 289 | 86.8% |  | 78.8% |  | 90.4% |  |
|  | | | | | | | |
| **0-4** | 203 / 224 | 94.1% | **.005** | 87.4% | **.005** | 93.0% | .187 |
| **5-6** | 216 / 249 | 85.9% |  | 77.4% |  | 89.3% |  |
|  | | | | | | | |
| **TV calc** | **Patients / Limbs** | **Limb salvage** | | **AFS** | | **Overall survival** | |
|  |  | Estimate | p-value | Estimate | p-value | Estimate | p-value |
| **0** | 158 / 173 | 92.9% | **.023** | 85.4% | **.034** | 91.7% | .706 |
| **1** | 60 / 67 | 95.5% |  | 89.5% |  | 93.3% |  |
| **2** | 201 / 233 | 85.8% |  | 77.6% |  | 90.0% |  |
|  | | | | | | | |
| **0-1** | 218 / 240 | 93.6% | **.007** | 86.6% | **.013** | 92.1% | .456 |
| **2** | 201 / 233 | 85.8% |  | 77.6% |  | 90.0% |  |

AFS = amputation-free survival. (m)PACSS = (modified) peripheral arterial calcium scoring system. TV calc = target vessel calcification.
NB. Both patients with and without technical success were included in this analysis.

**Supplementary Table 2. Technical success and primary patency rates distributed per PACSS, mPACSS or target vessel (TV) calcification groups.**

| **PACSS** | **Lesions TS / PP** | **Technical success** | | **Primary patency** | |
| --- | --- | --- | --- | --- | --- |
|  |  | Absolute | p-value | Absolute | p-value |
| **0** | 106 / 72 | 100 (94.3) | **< 0.001** | 64 (88.9) | .138 |
| **1** | 195 / 133 | 179 (91.8) |  | 103 (77.4) |  |
| **2** | 9 / 6 | 7 (77.8) |  | 5 (83.3) |  |
| **3** | 235 / 149 | 225 (95.7) |  | 123 (82.6) |  |
| **4** | 234 / 125 | 196 (83.8) |  | 93 (74.4) |  |
|  | | | | | |
| **0-2 (unilat)** | 310 / 211 | 286 (92.3) | .241 | 172 (81.5) | .464 |
| **3-4 (bilat)** | 469 / 274 | 421 (89.8) |  | 216 (78.8) |  |
|  | | | | | |
| **0,1,3 (< 5cm)** | 536 / 354 | 504 (94.0) | **< 0.001** | 290 (81.9) | .083 |
| **2,4 (≥ 5 cm)** | 243 / 131 | 203 (83.5) |  | 98 (74.8) |  |
|  | | | | | |
| **0-3** | 545 / 360 | 511 (93.8) | **< 0.001** | 295 (81.9) | .071 |
| **4** | 234 / 125 | 196 (83.8) |  | 93 (74.4) |  |
|  | | | | | |
| **mPACSS** | **Lesions TS / PP** | **Technical success** | | **Primary patency** | |
|  |  | Absolute | p-value | Absolute | p-value |
| **0** | 105 / 71 | 99 (94.3) | **.042** | 63 (88.7) | **.023** |
| **1** | 171 / 114 | 156 (91.2) |  | 84 (73.7) |  |
| **2** | 20 / 17 | 19 (95.0) |  | 16 (94.1) |  |
| **3** | 57 / 38 | 51 (89.5) |  | 32 (84.2) |  |
| **4** | 68 / 42 | 64 (94.1) |  | 30 (71.4) |  |
| **5** | 154 / 93 | 145 (94.2) |  | 81 (87.1) |  |
| **6** | 204 / 110 | 173 (84.8) |  | 82 (74.5) |  |
|  | | | | | |
| **0-2** | 296 / 202 | 274 (92.6) | .174 | 163 (80.7) | .747 |
| **3-6** | 483 / 283 | 433 (89.6) |  | 225 (79.5) |  |
|  | | | | | |
| **0-3** | 353 / 240 | 325 (92.1) | .252 | 195 (81.3) | .496 |
| **4-6** | 426 / 245 | 382 (89.7) |  | 193 (78.8) |  |
|  | | | | | |
| **0-4** | 421 / 282 | 389 (92.4) | .088 | 225 (79.8) | .890 |
| **5-6** | 358 / 203 | 318 (88.8) |  | 163 (80.3) |  |
|  | | | | | |
| **TV calc** | **Lesions TS / PP** | **Technical success** | | **Primary patency** | |
|  |  | Absolute | p-value | Absolute | p-value |
| **0** | 323 / 214 | 295 (91.3) | .415 | 169 (79.0) | .847 |
| **1** | 118 / 79 | 110 (93.2) |  | 63 (79.7) |  |
| **2** | 338 / 192 | 302 (89.3) |  | 156 (81.3) |  |
|  | | | | | |
| **0-1** | 441 / 293 | 405 (91.8) | .236 | 232 (79.2) | .578 |
| **2** | 338 / 192 | 302 (89.3) |  | 156 (81.3) |  |

Categorical outcomes are given as count (percentage).
(m)PACSS = (modified) peripheral arterial calcium scoring system. PP = primary patency. TS = technical success. TV calc = target vessel calcification.

**Supplementary Table 3. Odds ratios for technical failure at 3 months.**

|  | **Univariate** | | **Multivariable model**  PACSS 0-3 vs 4 | | **Multivariable model**  mPACSS 0-4 vs 5-6 | |
| --- | --- | --- | --- | --- | --- | --- |
|  | Odds ratio (95% CI) | P-value | HR (95% CI) | P-value | Hazard ratio (95% CI) | P-val |
| GLASS score (vs 1)  2  3  4 | **< 0.001**  2.66 (0.27 – 25.88) 0.400  8.38 (1.06 – 66.36) 0.044  24.38 (3.34 – 177.97) 0.002 | | < 0.001  3.40 (0.35 – 33.35) 0.400  11.80 (1.46 – 95.20) 0.044  34.33 (4.59 – 257.07) 0.002 | | < 0.001  3.70 (0.38 – 36.09) 0.261  13.53 (1.70 – 107.81) 0.014  41.03 (5.59 – 301.24) < 0.001 | |
| Location (vs popliteal)  Popliteal-tibial  Tibial | **< 0.001**  10.18 (3.16 – 32.74) < 0.001  4.88 (0.95 – 25.02) 0.057 | | < 0.001  15.76 (4.85 – 51.18) < 0.001  4.56 (0.88 – 23.73) 0.071 | | < 0.001  16.19 (4.98 – 52.59) < 0.001  4.61 (0.89 – 24.05) 0.069 | |
| PACSS 4 mPACSS ≥ 5 | 2.91 (1.78 – 4.76) **< 0.001**  1.53 (0.94 – 2.49) **0.088** | | 1.54 (0.91 – 2.62) 0.112 | | 1.38 (0.82 – 2.33) 0.226 | |

CI = confidence interval. GLASS = global limb anatomic staging system. (m)PACSS = (modified) peripheral arterial calcium scoring system.

|  | **Univariate** | | **Multivariable model**  PACSS 0-3 vs 4 | |
| --- | --- | --- | --- | --- |
|  | Odds ratio (95% CI) | P-value | HR (95% CI) | P-value |
| GLASS score (vs 1)  2  3  4 | **0.001**  1.44 (0.56 – 3.68) 0.446  1.63 (0.66 – 4.03) 0.292  3.43 (1.56 – 7.56) 0.002 | | 0.005  1.45 (0.56 – 3.81) 0.446  2.04 (0.79 – 5.22) 0.140  3.64 (1.54 – 8.59) 0.003 | |
| Location (vs popliteal)  Popliteal-tibial  Tibial | **0.001**  1.41 (0.84 – 2.37) 0.195  4.47 (2.00 – 10.00) < 0.001 | | 0.002  1.83 (1.06 – 3.15) 0.031  4.40 (1.92 – 10.09) < 0.001 | |
| DCB use | 0.71 (0.29 – 1.74) 0.454 | |  | |
| Stent use | 1.08 (0.60 – 1.94) 0.808 | |  | |
| PACSS 4 | 1.56 (0.96 – 2.53) 0.071 | | 1.10 (0.65 – 1.87) 0.729 | |

**Supplementary Table 4. Odds ratios for loss of primary patency at 3 months.**

CI = confidence interval. DCB = drug coated balloon. GLASS = global limb anatomic staging system. PACSS = peripheral arterial calcium scoring system.
